# Tunable Planar Hall Effect in (Ga,Mn)(Bi,As) Epitaxial Layers

**DOI:** 10.3390/ma14164483

**Published:** 2021-08-10

**Authors:** Tomasz Andrearczyk, Janusz Sadowski, Jerzy Wróbel, Tadeusz Figielski, Tadeusz Wosinski

**Affiliations:** 1 Institute of Physics, Polish Academy of Sciences, Aleja Lotnikow 32/46, PL-02668 Warsaw, Poland; andrea@ifpan.edu.pl (T.A.); sadow@ifpan.edu.pl (J.S.); wrobel@ifpan.edu.pl (J.W.); figiel@ifpan.edu.pl (T.F.); 2 Department of Physics and Electrical Engineering, Linnaeus University, SE-39182 Kalmar, Sweden

**Keywords:** dilute ferromagnetic semiconductors, molecular-beam epitaxy, planar Hall effect, magneto-crystalline anisotropy, spin–orbit coupling, spintronics

## Abstract

We have thoroughly investigated the planar Hall effect (PHE) in the epitaxial layers of the quaternary compound (Ga,Mn)(Bi,As). The addition of a small amount of heavy Bi atoms to the prototype dilute ferromagnetic semiconductor (Ga,Mn)As enhances significantly the spin–orbit coupling strength in its valence band, which essentially modifies certain magnetoelectric properties of the material. Our investigations demonstrate that an addition of just 1% Bi atomic fraction, substituting As atoms in the (Ga,Mn)As crystal lattice, causes an increase in the PHE magnitude by a factor of 2.5. Moreover, Bi incorporation into the layers strongly enhances their coercive fields and uniaxial magneto-crystalline anisotropy between the in-plane 〈110〉 crystallographic directions in the layers grown under a compressive misfit strain. The displayed two-state behaviour of the PHE resistivity at zero magnetic field, which may be tuned by the control of applied field orientation, could be useful for application in spintronic devices, such as nonvolatile memory elements.

## 1. Introduction

In the quest for more efficient spintronic materials, which are both useful for spintronic applications and interesting for physical phenomena investigations, we have focused on the quaternary (Ga,Mn)(Bi,As) compound [1,2]. The incorporation of a small amount of heavy Bi atoms into epitaxial layers of (Ga,Mn)As (regarded as the prototype dilute ferromagnetic semiconductor combining semiconducting properties with magnetism [3]) results in a strong enhancement of the spin–orbit interaction in the valence band, owing to a large relativistic correction to the band structure [4,5]. Our early measurements, performed by means of a modulation photoreflectance spectroscopy method [6], revealed that an addition of only 0.3% Bi atoms substituting As atoms in the (Ga,Mn)As crystal lattice caused a distinct modification of its valence band [1]. Moreover, it results in a significant increase in the magnitude of magnetotransport effects, as evidenced by our recent experiments performed for (Ga,Mn)(Bi,As) layers containing up to 1% Bi atomic fraction [7,8]. The enhanced spin–orbit interaction is especially favourable for spintronic materials as it enables one to electrically tune the magnetization direction through the spin–orbit torque effect [9].

The so-called planar Hall effect (PHE) is an interesting phenomenon arising due to the spin–orbit interaction in conducting ferromagnetic materials [10]. PHE consists of the development of a spontaneous transverse voltage in ferromagnetic layers with in-plane magnetization as a result of flowing longitudinal current. PHE resistivity in a single domain ferromagnetic layer can be described by the formula [11]:

*ρ_xy_* = ½(*ρ*_||_ − *ρ*_⊥_)sin2*θ*, (1)

where *ρ*_||_ and *ρ*_⊥_ are the resistivities for the in-plane magnetization vector oriented parallel and perpendicular to the current flow, respectively, and *θ* stands for the angle between the current direction and the magnetization vector. As found experimentally [12] and accounted for theoretically [13], in ferromagnetic (Ga,Mn)As layers (*ρ*_||_ − *ρ*_⊥_) < 0, which is the opposite to what is observed in metallic ferromagnets wherein *ρ*_||_ is mostly larger than *ρ*_⊥_. Moreover, Tang et al. [12] have shown that the magnitude of PHE is especially pronounced in (Ga,Mn)As layers. It is much larger than that in metallic ferromagnets, and the effect can be very useful in the investigations of magnetic properties of the layers. In the present paper, we report on our study of the planar Hall effect in (Ga,Mn)(Bi,As) epitaxial layers and its sensitivity to in-plane magnetic anisotropy.

## 2. Materials and Methods

We have investigated 50 nm thick (Ga,Mn)(Bi,As) layers, with 6% Mn and 1% Bi contents, grown on semi-insulating (001)-oriented GaAs substrate by the low-temperature molecular-beam epitaxy (LT-MBE) technique, at the substrate temperature of 230 °C. In addition, we have investigated similarly grown reference (Ga,Mn)As layers with 6% Mn and 50 nm thickness. After growth, the samples were subjected to a low-temperature annealing treatment carried out in air for 50 h at 180 °C. Long-term annealing thin (Ga,Mn)As layers at temperatures below the growth temperature results in a substantial improvement of magnetic and transport properties of the layers due to the out-diffusion of charge- and magnetic moment-compensating Mn interstitials [14,15]. Similar improvement was also established for the (Ga,Mn)(Bi,As) layers [1]. The in-depth composition of the annealed layers was analysed with secondary-ion mass spectrometry (SIMS) and shown elsewhere [2]. The high crystalline quality of the layers and sharp interfaces with the substrate were confirmed by means of high-resolution X-ray diffraction (XRD) and transmission electron microscopy (TEM) methods [8]. Structural characterization of the layers evidenced that both the (Ga,Mn)(Bi,As) and (Ga,Mn)As ones were grown pseudomorphically on GaAs substrate and suffer a biaxial compressive misfit strain. An incorporation of a small amount of Bi into the (Ga,Mn)As layers causes a distinct expansion of their lattice parameter perpendicular to the layer plane and an enhancement of the in-plane compressive strain [8].

The magnetic properties of the investigated layers were examined by means of superconducting quantum interference device (SQUID) magnetometry [2]. The incorporation of Bi into the (Ga,Mn)As layer resulted in a distinct broadening of its magnetization hysteresis loops and a decrease in the Curie temperature, which was about 90 K in the (Ga,Mn)(Bi,As) layer and 105 K in the reference (Ga,Mn)As one [2]. At low temperatures, both the (Ga,Mn)(Bi,As) and (Ga,Mn)As layers displayed the 〈100〉 in-plane easy magnetization axes and hard axes along two magnetically non-equivalent in-plane 〈110〉 crystallographic directions, wherein the [110] direction was noticeably harder than the [−110] perpendicular one. Such a magneto-crystalline anisotropy is typical for (Ga,Mn)As layers with sufficiently high hole concentration, grown under compressive misfit strain [16]. The origin of the intriguing uniaxial in-plane anisotropy along the 〈110〉 directions seems to be reliably explained as resulting from the preferred formation of Mn dimmers along the [−110] crystallographic direction at the (001) surface during the epitaxial growth of (Ga,Mn)As layers [17].

**Figure 1:**
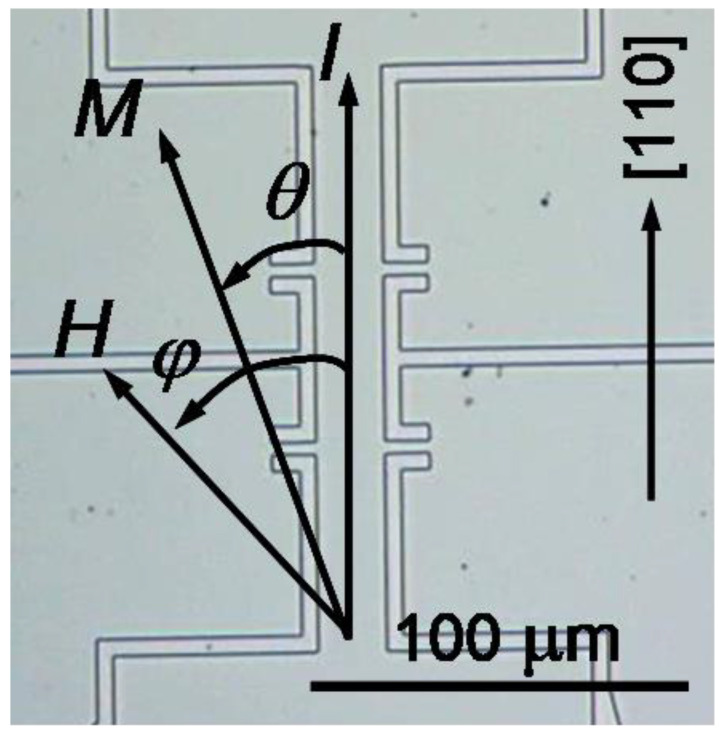
Microscopic image of a micro-Hall-bar tailored from the investigated (Ga,Mn)(Bi,As) layer along the [110] crystallographic direction and configuration for the PHE measurements. *θ* and *φ* denote angles between the current direction *I* and the magnetization vector *M* and applied in-plane magnetic field *H*, respectively.

PHE measurements have been performed with samples of micro-Hall-bar shape tailored from the investigated layers by means of electron-beam lithography patterning and chemical etching. Hall-bars of 20 μm width and 50 μm distance between the voltage contacts ensured an occurrence of single magnetic domains inside the bar [8]. A microscopic image of a micro-Hall-bar oriented along the [110] crystallographic direction of the (Ga,Mn)(Bi,As) layer and the measurement configuration are shown in Figure 1. The Hall-bars were supplied with Ohmic contacts to the (Ga,Mn)(Bi,As) and (Ga,Mn)As layers prepared by indium soldering to large contact areas located outside the image area. The PHE resistivity, *ρ_xy_*, has been measured, using a low-frequency lock-in technique and 0.5 μA sensing current, while sweeping an in-plane magnetic field, *H*, applied at various angles, *φ*, with respect to the current, *I*, flowing along the Hall-bar axis.

## 3. Results and Discussion

**Figure 2:**
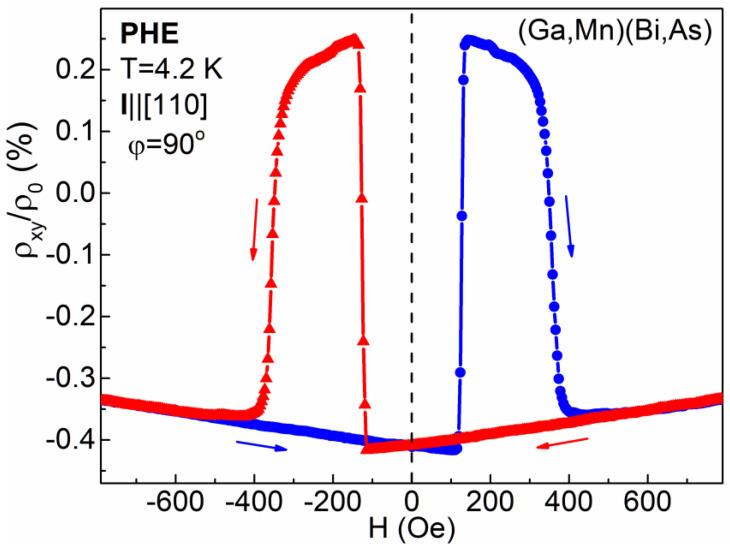
Normalized PHE resistivity for the (Ga,Mn)(Bi,As) micro-Hall-bar measured at the temperature 4.2 K, while sweeping an in-plane magnetic field, perpendicular to the current (*φ* = 90°), in opposite directions, as indicated by the arrows.

The up and down sweep of an in-plane weak magnetic field causes nonmonotonic changes of the PHE resistivity whose shape depends on the field orientation with respect to the Hall-bar axis. As an example, Figure 2 presents a record of changes of the PHE resistivity normalized to zero-field longitudinal resistivity, *ρ*_0_, measured for the (Ga,Mn)(Bi,As) layer at the liquid helium temperature under a magnetic field perpendicular to the Hall-bar axis, i.e., along the [−110] crystallographic direction at *φ* = 90°. The observed double-hysteresis-loop behaviour results from a magnetic-field-induced reorientation of the magnetization vector in the Hall-bar by 360° between the four equivalent easy axes oriented along the in-plane 〈100〉 crystallographic directions. While starting from the positive field value of about 600 Oe, the down and up field sweep causes a rotation of the magnetization vector, which assumes, in turn, the *θ* angles of 45°, 315°, 225°, 135° and again 45°, resulting in four transitions of the PHE resistivity between its low and high values. According to Equation (1), the low values of *ρ_xy_* = − ½(*ρ*_⊥_ − *ρ*_||_) appear at the *θ* angles of 45° and 225°, and the high values of *ρ_xy_* = ½(*ρ*_⊥_ − *ρ*_||_) appear at the *θ* angles of 135° and 315°. The difference between the high and low values corresponds to the amplitude of PHE resistivity and equals *ρ*_⊥_ − *ρ*_||_. Qualitatively similar behaviour has been registered for the Hall-bar of the reference (Ga,Mn)As layer, displaying, however, significantly smaller amplitude of the PHE resistivity changes and the transitions between their low and high values occurring at much smaller values of magnetic field, corresponding to smaller values of coercive fields in (Ga,Mn)As; c.f. [7].

**Figure 3:**
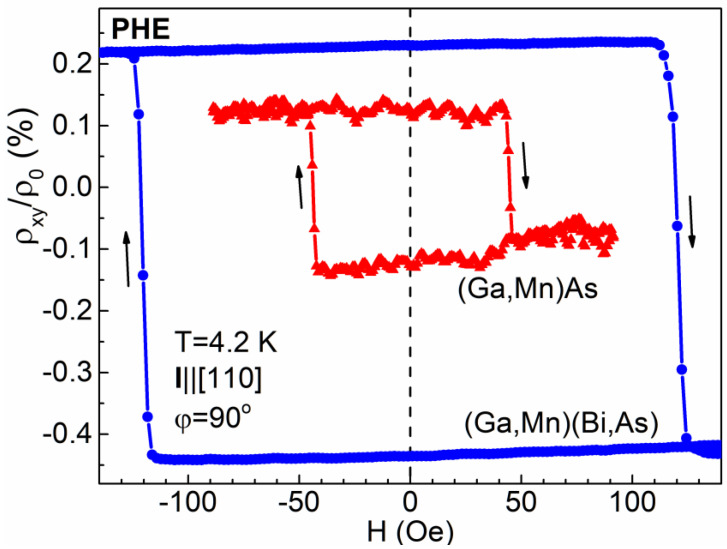
Same as in Figure 2 but measured for a narrower range of sweeping magnetic field after previously magnetizing the Hall-bar at the positive field of 600 Oe. Results obtained for a Hall-bar of the reference (Ga,Mn)As layer are also shown for comparison.

The PHE resistivity record, obtained while sweeping the magnetic field in a narrower range of about ±150 Oe, corresponding to the field values between the up and down resistivity transitions, displays a single-hysteresis-loop dependence, as shown in Figure 3. Here, while starting from the positive field value, the field sweep causes a magnetization vector rotation by 90° between two in-plane easy axes at the *θ* angles of 45° and 315° and back to 45°. Similar record obtained for the reference (Ga,Mn)As layer is also shown in Figure 3. It presents a single hysteresis loop of the PHE resistivity, which is by a factor of about 2.7 narrower and displays the amplitude of normalized PHE resistivity, (*ρ*_⊥_ − *ρ*_||_)/*ρ*_0_, by a factor of about 2.5 smaller than that for the (Ga,Mn)(Bi,As) layer.

It is worth noting that the PHE resistivity record registered while sweeping a magnetic field may depend on the uncontrolled misalignment of the exact field orientation; c.f. [18]. The results presented in Figure 2 and Figure 3 correspond to the *φ* angle slightly smaller than 90° and caused by the magnetization vector rotations in the clockwise direction. For the field oriented at the *φ* angle slightly larger than 90° the shape of PHE resistivity scan would display the reversed polarity, resulting from rotations of the magnetization vector inside the Hall-bar in the counter-clockwise direction. Nevertheless, in that case, the transitions between the high and low values of PHE resistivity occur at the same field values, as those shown in Figure 2 and Figure 3.

**Figure 4:**
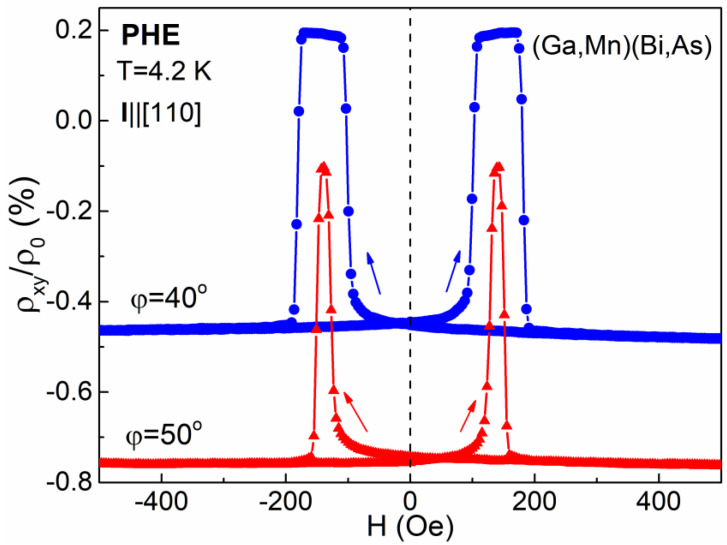
Same as in Figure 2 but measured for the sweeping magnetic field applied at the *φ* angle values of 40° and 50°. The curves are vertically offset for clarity.

**Figure 5:**
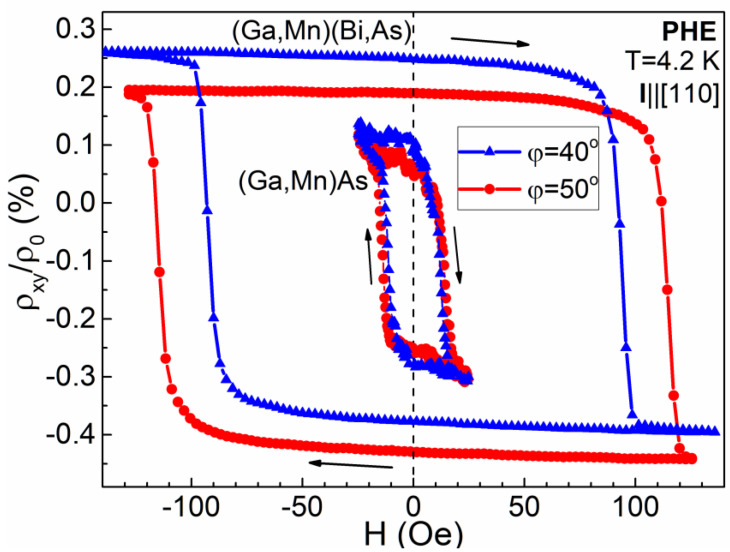
Same as in Figure 3 but measured for the sweeping magnetic field applied at the *φ* angle values of 40° and 50°. The curves for the (Ga,Mn)(Bi,As) Hall-bar are vertically offset for clarity.

Intriguingly, much more spectacular dependence on misalignment of the field orientation with respect to the exact crystallographic direction occurs in the case of a field applied along the easy magnetization axes. In Figure 4 and Figure 5, we present the PHE resistivity records measured while sweeping the field applied at the *φ* angle values of 40° and 50°, i.e., the values of 5° smaller and 5° larger, respectively, with respect to the one corresponding to the [10] easy axis. In the case of *φ* = 40°, the rotation of magnetization vector in the Hall-bar by 360° occurs in the counter-clockwise direction, while for *φ* = 50° that rotation occurs in the opposite, clockwise direction. In the former case, when starting from the positive field value, the first transition of the magnetization vector between the easy axes occurs by overcoming the [−110] crystallographic direction, while in the latter case it occurs by overcoming the magnetically harder [110] one, thus requiring a distinctly larger value for the coercive field, as shown in Figure 4. Consequently, the single hysteresis loops, obtained while sweeping a magnetic field in a narrower range (Figure 5), differ significantly in their width depending on the applied *φ* angle. For the (Ga,Mn)(Bi,As) layer, the width of the PHE resistivity hysteresis loop recorded at *φ* = 50° becomes about 25% larger than that recorded at *φ* = 40°. For the reference (Ga,Mn)As layer, the respective difference is smaller and amounts to about 15%. On the other hand, for the *φ* angle close to 90° (Figure 2 and Figure 3), the magnetization vector rotation between the easy axes occurs by overcoming the harder axis, of either along the same [110] or opposite [−1−10] direction, dependent on the *φ* angle misalignment.

Rigid transitions between the low and high PHE resistivities in the (Ga,Mn)(Bi,As) micro-Hall-bars confirm the occurrence of single magnetic domains in the Hall-bars. This is in agreement with the previous results of a scanning SQUID microscope [19] and magneto-optical domain imaging [20], which provided evidence of the presence of large magnetic domains of hundreds-of-micrometers dimensions in (Ga,Mn)As layers with in-plane magnetization. Magnetization vector rotation between the easy axes in those layers occurred through the nucleation and propagation of a 90° domain wall existing over a narrow magnetic field range in the vicinity of the coercive field [20]. Importantly, our results show that a substitution of 1% As atoms by heavy Bi atoms in (Ga,Mn)As layers results (owing to increased spin–orbit interaction) in a significant enhancement of the magnitude of normalized PHE resistivity by a factor of 2 to 3, as obtained for a set of several micro-Hall-bars and various orientations of the in-plane magnetic field.

## 4. Conclusions

Our investigations of the planar Hall effect in (Ga,Mn)(Bi,As) layers grown under a compressive misfit strain confirm the presence of the in-plane magneto-crystalline anisotropy with the cubic 〈100〉 easy axes and magnetically non-equivalent 〈110〉 hard axes at low temperatures, qualitatively similar to that present in (Ga,Mn)As layers. However, a small addition of heavy Bi atoms to (Ga,Mn)As results in significantly larger coercive fields and distinctly stronger uniaxial anisotropy between the perpendicular 〈110〉 crystallographic directions. The incorporation of just 1% Bi into the (Ga,Mn)As layer causes an essential enhancement of the amplitude of PHE resistivity by a factor of 2 to 3, as a result of the increased strength of the spin–orbit coupling. The two-state behaviour of the PHE resistivity at zero magnetic field, which may be tuned by the applied field orientation, provides its usefulness for applications in nonvolatile memory elements. The quaternary (Ga,Mn)(Bi,As) ferromagnetic semiconductor appears also to be a promising material for studying the operation of novel spintronic devices, such as the recently proposed magnetoelectric spin–orbit logic device [21], requiring the high-spin–orbit-coupling materials.

## Data Availability

The data presented in this study are available on request from the corresponding author.
